# Dermatologic toxicities related to cancer immunotherapy

**DOI:** 10.1016/j.toxrep.2025.102021

**Published:** 2025-04-05

**Authors:** Yosra Vaez-Gharamaleki, Mohammad Amin Akbarzadeh, Farhad Jadidi-Niaragh, Ata Mahmoodpoor, Sarvin Sanaie, Mohammad-Salar Hosseini

**Affiliations:** a Hematology and Oncology Research Center, Tabriz University of Medical Sciences, Tabriz, Iran; b Research Center for Evidence-Based Medicine, Iranian EBM Center: A JBI Center of Excellence, Tabriz University of Medical Sciences, Tabriz, Iran; c Immunology Research Center, Tabriz University of Medical Sciences, Tabriz, Iran; d Department of Immunology, Faculty of Medicine, Tabriz University of Medical Sciences, Tabriz, Iran; e Department of Anesthesiology and Critical Care Medicine, Faculty of Medicine, Tabriz University of Medical Sciences, Tabriz, Iran; f Research Center for Integrative Medicine in Aging, Aging Research Institute, Tabriz University of Medical Sciences, Tabriz, Iran; g Iranian Cancer Control Center (MACSA) – Tabriz Branch, Tabriz, Iran

**Keywords:** Cutaneous adverse events, Drug-related side effects and adverse reactions, Immune checkpoint inhibitors, Immunotherapy, Immune-related adverse events, Immune-related toxicities, Palliative care

## Abstract

Immunotherapy has revolutionized cancer treatment, offering significant survival superiority for advanced malignancies. However, immunotherapy is associated with various immune-related adverse events, one of the most common of them being dermatologic toxicities. Previous studies have reported dermatologic adverse events in almost half of the cancer patients undergoing immunotherapy. The spectrum of dermatologic toxicities ranges from mild, self-limiting reactions to severe, life-threatening conditions, and includes maculopapular rash, pruritus, vitiligo-like depigmentation, psoriasiform eruption, lichenoid eruption, bullae, photosensitivity, hair loss, nail changes, Stevens-Johnson syndrome, and toxic epidermal necrolysis. The management strategies are based on personalized treatment plans, multidisciplinary approaches, and timely therapeutic interventions aimed at addressing dermatologic toxicities while preserving immunotherapy efficacy. Based on the latest findings, this paper offers a novel perspective and provides an evidence-based review of the pathogenesis, manifestations, incidence, grading, clinical management, and prognostic significance of these toxicities, underlining the importance of balancing the efficacy of immunotherapy with timely and proactive management of their dermatological toxicities to enhance patient outcomes and quality of life.

## Highlights

• Dermatologic toxicities are one of the most common irAEs, with almost 50 % incidence in immunotherapy-treated cancer patients.
• Immune-related dermatologic toxicities range from mild rashes to severe, life-threatening conditions such as SJS/TEN.
• T-cell hyperactivation and immune dysregulation underlie most immune-related skin toxicities.
• Management strategies vary by toxicity grade, with early detection, symptom relief, and immunosuppressive therapies as basis.
• Future studies should focus on biomarker-driven precision medicine and address immunogenetic risk factors for toxicity prevention.

## Graphical Abstract

**Figure:**
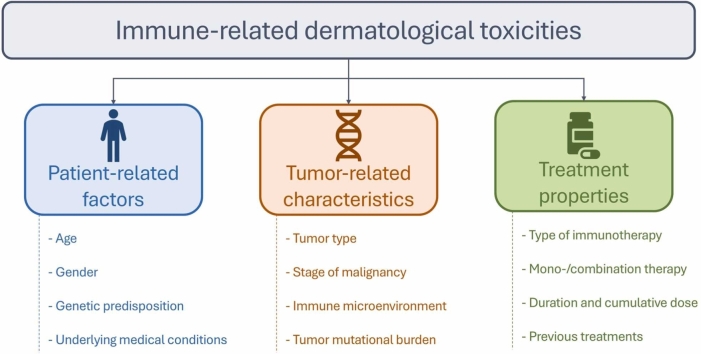

## 1 Introduction

Cancer immunotherapy represents a transformative shift in cancer treatment. Unlike conventional therapies, such as chemotherapy and radiation therapy, where clinical toxicity and treatment resistance are frequently observed, immunotherapy offers a targeted and potentially durable response [1]. Key discoveries, such as the immune checkpoints and their role in cancer progression, have ultimately led to novel interventions with durable responses and improved clinical outcomes, such as the inhibitors of cytotoxic T-lymphocyte associated protein-4 (CTLA-4), the programmed cell death-1 (PD-1) and its ligand (PD-L1), and lymphocyte activation gene-3 (LAG-3) [2]. Immunotherapy modalities disrupt tumor immune evasion, promoting potent antitumor responses and durable remissions in specific cancers [3]. The concept of combination immunotherapy has led to the development of various therapeutic protocols involving different types of immunotherapies, including monoclonal antibodies, immune system modulators, cancer vaccines, adoptive cell transfer, and chimeric antigen receptor (CAR)-T cell therapy [4]. However, challenges such as primary and acquired resistance, acute or delayed adverse events, and the lack of effective prognostic biomarkers underline the complexity of immune modulation.

The importance of immune-related adverse events (irAEs), specifically dermatologic toxicities of immunotherapy, lies in their prevalence, clinical impact, and management challenges. Latest studies have reported dermatologic toxicities occurring in one-third to over half of the cancer patients receiving single-agent or combined regimens of immune checkpoint inhibitors (ICIs), with severity ranging from mild rash to life-threatening conditions such as toxic epidermal necrolysis (TEN) [5], [6]. Moreover, large-scale studies have estimated a 1.5- to 4-fold increase in the incidence of adverse events following immunotherapy, with various factors such as gender, antibiotic use, body mass index, and baseline laboratory values proposed as predictors of irAEs’ occurrence and severity [7], [8]. These toxicities not only affect patients’ quality of life but may also induce treatment interruptions or discontinuations, thereby influencing treatment outcomes. Moreover, dermatologic toxicities could be potentially used as clinical indicators of underlying immune activation and treatment response [9]. Recent studies have associated the occurrence of certain dermatologic toxicities, such as vitiligo-like lesions, with improved treatment outcomes, reflecting systemic immune activation against tumor antigens [10].

Considering the ever-growing importance of immunotherapy, this review provides deeper insights into the mechanisms, management, and future directions of immunotherapy-related dermatological toxicities, aiming to improve clinical practice and patient-centered outcomes.

## 2 Methods

Following an evidence-based formulated research question [11], MEDLINE (via PubMed), Scopus, Web of Science, and the Cochrane Library were searched with free keywords and MeSH terms representing cancer, immunotherapy, and clinical cutaneous manifestation, using a broad and highly sensitive search strategy in order to maximize the inclusion. Key records were obtained regardless of language and time of publication until the end of 2024, and screened based on their relevance to the main topic. Studies addressing the dermatological toxicities related to cancer immunotherapy were selected, and irrelevant records, *in vitro* studies, study protocols without results, and studies without reported dermatological toxicities were excluded. Two authors independently assessed the studies for potential biases, with points of discrepancies resolved through discussion. The ultimately-included studies were reviewed, and all relevant information was gathered, categorized, and presented using narrative synthesis.

## 3 Pathogenesis

The precise pathogenesis and underlying mechanisms of immune-related dermatological toxicities have not been thoroughly identified. Immunotherapy-related dermatologic toxicities involve complex interactions between the immune system, tumor microenvironment, and cutaneous tissues. Table 1 presents an outline for the immunopathogenesis of immune-related dermatological toxicities.

**Table 1:** Overview of the underlying immunopathogenesis and core components of the immune-related dermatological toxicities.

Immunopathogenesis	Key components	Mechanisms of action	Associated dermatological presentation
T-cell activation and dysregulation	Th1, Th17, and Immune checkpoints such as PD-1/PD-L1 and CTLA-4	Hyperactivation of T-cells, leading to autoreactive T-cell proliferation and tissue damage, cross-reactivation with skin antigens	Maculopapular rash, vitiligo-like depigmentation, lichenoid dermatitis
Cytokine and chemokine signaling	IFN-γ, TNF-α, IL-6, IL-17, IL-23, CXCL10	Pro-inflammatory response, recruitment of immune cells to the skin, and keratinocyte apoptosis	Rash, psoriasiform eruption, pruritus
Antibody-mediated effects	Autoantibodies	Production of autoantibodies against skin components, causing autoimmunity and direct tissue injury.	Bullous dermatoses
Microbiome dysregulation	Skin microbiota	Dysbiosis alters skin immune homeostasis, increasing susceptibility to inflammation and infection	Folliculitis, erythema, secondary infections
Dendritic cell activation	Antigen-presenting dendritic cells, costimulatory molecules (CD80/CD86)	Enhanced antigen presentation amplifies immune activation, leading to heightened skin immune responses	Rash, psoriasiform eruption, lichenoid dermatitis

**Abbreviations:***CD:* Cluster of differentiation. *CTLA-4:* Cytotoxic T-lymphocyte associated antigen-4. *CXCL10:* C-X-C motif chemokine ligand 10. *IFN-γ:* Interferon-gamma. *IL:* Interleukin. *PD-1/PD-L1:* Programmed cell death protein-1 and its ligand. *Th:* T helper. *TNF-α:* Tumor necrosis factor-alpha.

Aberrant immune activation is the cornerstone of immunotherapy-related dermatological toxicities. ICIs inhibit regulatory pathways that suppress T-cell activity, restoring an effective antitumor immune response. However, this immune reactivation can inadvertently target self-tissues, including the skin. In this scenario, self-antigens expressed on keratinocytes, melanocytes, or other skin components become unintended targets of activated T-cells [12]. The key driver in this case is the overactivation of T-helpers, such as Th1 and Th17 cells [13], [14]. The release of pro-inflammatory cytokines promoting tissue inflammation, along with their contribution to keratinocyte proliferation abnormalities and disruption of skin integrity, aggravates the inflammatory cascade endpoints.

Cytokine-mediated inflammation is another outcome of the post-immunotherapy aberrant immune activation. The immune activation triggered by immunotherapy leads to the release of pro-inflammatory cytokines, damaging epidermis and dermis. Tumor necrosis factor-alpha (TNF-α) is a chief mediator that amplifies inflammatory responses in the dermis and epidermis, resulting in irAEs such as erythema and pruritus [15]. Interleukin-6 (IL-6) plays a dual role, exacerbating both local and systemic inflammations [16]. Interferon-gamma (IFN-γ) drives keratinocyte apoptosis and compromises melanocyte survival, explaining conditions such as vitiligo [17]. Additionally, interleukin-17 (IL-17) and interleukin-23 (IL-23), which are central to psoriasiform dermatitis by disrupting keratinocyte proliferation and barrier function, may also lead to the characteristic scaly plaques observed in affected patients [18], [19].

The development of autoimmune reactions against self-antigens expressed in the skin is another proposed mechanism for immune-related dermatologic toxicities [20]. Immunotherapy triggers the activation of autoreactive T-cells and B-cells, producing autoantibodies targeting components of the epidermis and dermis. For instance, in ICI-induced bullous pemphigoid, autoantibodies against BP180 and BP230 antigens have been detected, resulting in immune-mediated blister formation [21], [22]. The pathogenesis of autoimmune reactions against skin antigens involves both humoral and cellular immune responses. Autoreactive T-cells recognize and attack antigen-presenting cells, presenting skin-derived antigens, leading to the activation of B-cells and the production of autoantibodies. Additionally, pro-inflammatory cytokines released by activated immune cells contribute to tissue damage and inflammation within the skin. As discussed later, genetic predisposition plays a significant role in developing autoimmune reactions against skin antigens. Environmental triggers, such as medications, infections, and ultraviolet radiation, may also precipitate or exacerbate autoimmune skin diseases in genetically susceptible individuals [23].

Immune checkpoints such as PD-1 and CTLA-4 are naturally expressed on skin-resident T-cells to maintain local immune tolerance [24]. ICIs block these checkpoints, disrupting the delicate immune homeostasis of the skin through persistent T-cell activation. Furthermore, emerging research highlights the potential involvement of the skin microbiome in immunotherapy-related dermatological toxicities [25]. Dysbiosis may predispose individuals to heightened inflammation and autoimmune reactions during immunotherapy [26]. Microbial antigens can trigger immune activation, particularly in genetically susceptible individuals. Although the exact role of the skin microbiome in these toxicities remains under investigation, it represents a promising area for future therapeutic interventions, including microbiome-modulating strategies.

## 4 Clinical presentations

Dermatological complications appear in various forms and presentations, ranging from simple rash to life-threatening epidermal necrolysis. Some irAEs, such as rash, are more common, while some others, such as the TEN – although less common – present with a significantly higher health burden, requiring clinical vigilance for the earliest signs of dermatological toxicities in patients undergoing immunotherapy [27]. Maculopapular rashes are the most commonly observed cutaneous irAEs [27], [28]. Usually early-onset, these rashes result from early immunological response to treatment and are most common in patients receiving anti-CTLA-4 immunotherapies and combination therapy regimens [29], [30]. The morphology and severity of the rash can vary widely between patients and may evolve over time. Common presentations include erythematous or eczematous patches, papules, vesicles, and desquamation. Primarily involving the trunk and extremities, the rash is sometimes accompanied by mild pruritus [31]. While generally mild, severe cases may require systemic interventions.

Pruritus may occur with or without visible skin changes, making it a diagnostically challenging presentation [32]. Although it almost always presents in non-severe forms, it significantly affects the patients’ quality of life. The pruritus is potentially mediated by cytokines, including IL-6 and TNF-α, which are upregulated during immune activation [33]. Hypopigmentation is another distinct immune-mediated dermatological toxicity observed in patients undergoing immunotherapy. It represents the autoimmune destruction of melanocytes, leading to depigmented macules and patches. Studies have reported that vitiligo-like hypopigmentation, or in some cases, depigmentation, is strongly associated with favorable clinical outcomes, as its development suggests robust immune activation against melanocyte-associated antigens shared by tumor cells [34]. These changes, however, generally tend to persist, although there have been few reports of reversal [35].

Psoriasiform eruptions, presented with erythematous plaques and overlying silvery scales, may occur de novo or as an exacerbation of pre-existing psoriasis [36]. It is often associated with Th17-mediated immune responses, which are augmented by ICIs [37]. Patients may present with lesions on extensor surfaces, such as elbows and knees, or with scalp involvement. Bullous dermatoses, though rare, are a significant irAE due to their potential morbidity. They are characterized by subepidermal blistering resulting from autoantibody formation against hemidesmosomal proteins, specifically BP180 and, less commonly, BP230 [38]. Clinically, it presents with tense blisters on erythematous or urticarial skin, typically sparing mucosal surfaces. Histological examination reveals a subepidermal blister with eosinophilic infiltration, and direct immunofluorescence confirms the presence of IgG and C3 deposition along the basement membrane zone [39]. Most patients exhibit a series of prodromal symptoms beforehand, the most common being erythema and pruritus [40].

The most severe dermatological irAEs are Stevens-Johnson syndrome (SJS) and TEN, both of which are rare but life-threatening. Characterized by widespread epidermal necrosis and detachment, both adverse events are accompanied by mucosal involvement. The initial symptoms may include fever, malaise, and a painful rash that progresses to bullae and extensive skin sloughing [41]. Histologically, there is full-thickness epidermal necrosis and minimal dermal lymphocytic infiltrate [42], [43].

Other presentations are less common, but there have been reports of eosinophilic fasciitis and photosensitivity [44], [45], [46]. Neutrophilic dermatoses like Sweet’s syndrome are rare conditions that may be caused by anti-CTLA-4 and PD-1 therapy [47], [48]. They are mostly presented by granuloma annulare and sarcoidosis. Immunotherapy can also be associated with rosaceiform eruptions, lupus, dermatomyositis and several kinds of vasculitis [47], [49]. Mucosal irAEs are observed in many patients, including xerostomia, mucositis, and dysgeusia [50], [51]. Alopecia and nail changes, including color changes and nail loss, may also be observed in some cases [52], [53]. Table 2 presents an overview of the most common dermatological toxicities related to cancer immunotherapy.

**Table 2:** A detailed overview of the most common dermatological adverse events following cancer immunotherapy.

Dermatological toxicities	Incidence	Typical clinical presentation	Onset of presentation	Duration	Histopathology	Potential long-term effects
Rash, maculopapular eruption	High (around one-third to half of patients)	Erythematous macules or papules, often pruritic	Early-onset, usually within the first month	Weeks to months	Lymphocytic infiltration and spongiosis	Generally resolving without sequelae
Pruritus	High (around one-third)	Generalized itching, with or without rash	Variable (early or anytime during therapy)	Variable – may persist if untreated	Minimal or no specific changes	Chronic discomfort may persist in some cases
Vitiligo-like leukoderma	Common	Hypopigmented or depigmented macules and patches	Variable (weeks to months)	Generally persistent	Loss of melanocytes	Irreversible in most patients
Psoriasiform eruption	Relatively common	Erythematous plaques with silvery scaling	Early-onset, usually within the first couple of months	Persistent if untreated – chronic course possible	Acanthosis, parakeratosis, and lymphocyte infiltration	Scarring, prone to secondary bacterial infections
Lichenoid eruption	Relatively common	Flat-topped papules	Early-onset, usually within the first couple of months	May persist or improve with treatment discontinuation	Dense and band-like lymphocytic infiltration in dermo-epidermal junction with basal layer damage	Risk of chronic hyperpigmentation and lichen scarring in some cases
Hair loss and alopecia	Less common*	Diffuse or patchy hair loss	Delayed-onset in most cases	Permanent in some cases	Peribulbar lymphocytic infiltration	Risk of persistent hair loss, associated with psychosocial impact
Bullous dermatoses	Less common	Tense blisters on erythematous or urticarial skin	Late-onset	Months to years, often requiring prolonged treatment	Subepidermal blistering with eosinophilic infiltration	Associated with skin fragility and scarring
Nail changes	Less common*	Discoloration, ridging, brittleness, or loss of nails	Late-onset	Variable – may persist long-term	Periungual inflammation, keratinocyte damage in severe cases	Chronic nail dystrophy and functional impairment in severe cases
Photosensitivity	Rare	Increased sensitivity to light, in the form of erythema or even blistering	Variable (weeks to months)	Variable – depends on exposure and duration of treatment	Nonspecific, but necrotic keratinocytes, dyskeratosis, dermal edema, and mixed infiltration may be observed	Risk of chronic photosensitivity
SJS/TEN	Rare	Widespread epidermal detachment, bullae formation, and mucosal involvement	Acute-onset	Weeks to months – depending on recovery in intensive care	Full-thickness epidermal necrosis, sparse dermal inflammation	Scarring, pigmentation changes, chronic pain

* more common with other anticancer treatment modalities. **Abbreviation:***SJS:* Stevens-Johnson syndrome. *TEN:* Toxic epidermal necrolysis.

## 5 Grading and clinical spectrum

Grading dermatological irAEs is standardized using the Common Terminology Criteria for Adverse Events (CTCAE) to ensure uniformity in reporting and clinical management [54]. The grading system typically classifies toxicities from Grade 1 (mild) to Grade 5 (death), reflecting the severity and functional impact of the adverse event. Grade 1 dermatological toxicities are usually limited to asymptomatic or mild symptoms, such as localized rash or pruritus, without functional impairment. Grade 2 indicates minimal or moderate symptoms that may interfere with activities of daily living but do not require hospitalization and usually respond well to local interventions. Grade 3 toxicities are severe or clinically significant, causing functional impairment, extensive skin involvement, or requiring systemic therapy or hospitalization. Grade 4 represents life-threatening conditions such as SJS or TEN, often requiring intensive care and immediate interventions, such as the cessation of immunotherapy. Grade 5, although rare, is defined as adverse event-related fatal outcomes. Table 3 summarizes the grading of the most common immune-related dermatological toxicities based on the Common Terminology Criteria for Adverse Events (CTCAE) version 5.0.

**Table 3:** Grading of common cutaneous immune-related adverse events. The grading is based on the Common Terminology Criteria for Adverse Events (CTCAE) v5.0, with higher grades indicating more severe adverse events. Grade 1 indicates mild, grade 2 indicates moderate, grade 3 indicates severe, and grade 4 indicates life-threatening toxicities.

Dermatological manifestations	Definition	CTCAE Grading				
		Grade 1	Grade 2	Grade 3	Grade 4	Grade 5
Alopecia	Reduced hair density relative to what is typical for a person at the certain age and body location	- Less than 50 % hair loss - Not obvious from a distance	- Over 50 % - Associated psychosocial impact	ND	ND	ND
Bullous dermatitis	Inflamed skin with fluid-filled bullae present	- Asymptomatic - Blisters covering less than 10 % BSA	- Painful blisters - Blisters covering 10–30 % BSA - Limited iADL	- Blisters covering over 30 % BSA - Limited scADL	- Blisters over 30 % BSA - Fluid/electrolyte abnormalities present - Critical care indicated	Death
Nail changes	Changes in nails, color of nail plates, or loss of all/portion of the nail	- Asymptomatic discoloration or nail bed-plate separation	- Symptomatic nail bed-plate separation - Limited iADL	ND	ND	ND
Photosensitivity	Increased skin sensitivity to light	- Painless erythema covering less than 10 % BSA	- Tender erythema covering 10–30 % BSA	- Erythema covering over 30 % BSA and erythema with blistering - Corticosteroid/analgesic indicated	- Life-threatening Consequences - Urgent Intervention indicated	Death
Pruritus	Intense itching sensation	- Mild or localized - Topical intervention indicated	- Widespread and intermittent - Skin changes from scratching (edema, papulation, excoriations, lichenification, oozing/crusts) - Local intervention indicated - Limited iADL	- Widespread and constant - Limited scADL/sleep - Systemic intervention indicated	ND	ND
Purpura	Discolored red or purple hemorrhagic areas on skin and mucous membrane	- Combined coverage of less than 10 % BSA	- Combined coverage of 10–30 % BSA - Bleeding with trauma	- Combined coverage of over 30 % BSA - Spontaneous bleeding	ND	ND
Rash and maculopapular eruption	Presence of macules and papules	- Covering less than 10 % BSA, regardless of the presence of other symptoms	- Covering 10–30 % BSA, regardless of the presence of other symptoms - Limited iADL	- Covering over 30 % BSA with moderate/severe symptoms - Limited scADL	ND	ND
Skin hypopigmentation	Hypopigmentation or depigmentation, as observed in vitiligo and vitiligo-like leukoderma	- Covering less than 10 % BSA	- Covering over 10 % BSA - Associated psychosocial impact	ND	ND	ND
SJS	Less than 10 % total body skin area separation of dermis	ND	ND	- Skin sloughing covering less than 10 % BSA with associated signs (such as erythema, purpura, and epidermal or mucous membrane detachment)	- Skin sloughing covering 10–30 % BSA with associated signs	Death
TEN	Over 30 % total body skin area separation of dermis	ND	ND	ND	- Skin sloughing covering 30 % or more of BSA, with associated symptoms (such as erythema, purpura, or epidermal detachment)	Death

**Abbreviations:***BSA:* Body surface area. *iADL:* Instrumental activities of daily living. *ND:* Not defined. *scADL:* Self-care activities of daily living. *SJS:* Stevens-Johnson syndrome. *TEN:* Toxic epidermal necrolysis.

**Table 4:** Summary of the recent and key clinical studies reporting dermatological toxicities following cancer immunotherapy.

Authors	Year	Country	Type of immunotherapy	Cancer type	Type of cutaneous toxicities	Severity and frequency
Zhang et al. [118]	2023	China	CAR T-cell	T-cell ALL	Maculopapular rash, GVHD-like skin reaction	Grade 1–2 cutaneous irAEs (overall frequency: 20 %)
Tan et al. [119]	2023	China	CAR T-cell	T-cell ALL	GVHD-like skin reaction	Various grades (overall frequency: 80 %), one being grade 3–4
Le et al. [120]	2024	USA	ICI (various), monoclonal antibody	Various cancers	Various toxicities (most common lichenoid, eczematous, and psoriasiform dermatitis)	Various grades, the most common being grade 2 (66 %), with one case of grade 4 toxicity
Juan-Carpena et al. [121]	2024	Spain	ICI (various)	Various cancers	Various toxicities (most common pruritus, eczema, and maculopapular rash)	Various grades of dermatological irAEs (overall frequency: 43.4 %), the most common being grades 1–2 (94 %)
Wan et al. [122]	2024	USA	ICI (various)	Various cancers	Various toxicities (most common rash, pruritus, and skin hypersensitivity)	Cutaneous irAEs (overall frequency: 25 %) had significant co-occurrence with all other irAEs
Nardin et al. [123]	2022	France	ICI (anti-PD−1)	Melanoma	Vitiligo	Vitiligo was reported in overall of 13.5 % of the patients
Min Lee et al. [124]	2018	USA	ICI (anti-PD−1)	Various cancers	Dermatitis	Dermatitis was most frequent in cutaneous malignancies (43 %) and head and neck cancers (20 %)
Nelson et al. [125]	2022	USA	ICI (anti-PD−1)	Various cancers	Bullous pemphigoid	Bullous pemphigoid was most frequent in NSCLC (33 %), melanoma (25 %), and non-melanoma skin cancer (17 %)
Thiruvengadam et al. [126]	2020	USA	ICI, CAR T-cell	DLBCL	GVHD-like skin reaction	Grade 3 cutaneous irAE (overall frequency: 8.3 %)
Boude et al. [127]	2016	Netherlands	Dendritic cell vaccination	Melanoma	Injection Site reactions	Grade 1 cutaneous irAE (overall frequency: 50 %)
Yoshida et al. [128]	2011	Japan	Personalized peptide vaccine	Pancreatic cancer	Cellulitis and ulceration in injection site	Both cutaneous irAEs were CTCAE grade 3 (overall frequency: 0.4 %)
Chan et al. [129]	2020	USA	ICI (anti-PD−1)	Lung adenocarcinoma, Metastatic melanoma	Eosinophilic fasciitis	Grade 3 cutaneous irAEs in all reported patients
Pintova et al. [130]	2013	USA	ICI (anti-CTLA−4)	Metastatic melanoma	Neutrophilic dermatosis (Sweet’s syndrome)	Grade 2 cutaneous irAE in the reported patient
Moehler et al. [131]	2019	Germany	Oncolytic virus (Pexastimogene devacirepvec, Pexa-Vec)	HCC	Papulopustular rash	Grade 1 cutaneous irAEs (overall frequency: 29 %)
Chesney et al. [132]	2018	USA	Oncolytic virus (Talimogene laherparepvec) + ICI (anti-CTLA−4)	Melanoma (stages III-IV)	Rash and pruritus	Grade 1 cutaneous irAEs (overall frequency: 39 %)
Parisi et al. [133]	2023	USA	Monoclonal antibody (Blinatumomab)	B-cell ALL	Acne, rash, nail changes, erythema, psoriasis, and seborrheic dermatitis	Grade 1–2 cutaneous irAEs (overall frequency: 15 %)

**Abbreviations:***ALL:* Acute lymphoblastic leukemia. *CAR:* Chimeric antigen receptor. *CTCAE:* Common Terminology Criteria for Adverse Events. *CTLA-4:* Cytotoxic T-lymphocyte associated antigen-4. *DLBCL:* Diffuse large B-cell lymphoma. *GVHD:* Graft-versus-host disease. *HCC:* Hepatocellular carcinoma. *ICI:* Immune checkpoint inhibitor. *irAE:* Immune-related adverse events. *NSCLC:* Non-small cell lung cancer. *PD-1:* Programmed cell death protein-1. *SJS:* Stevens-Johnson syndrome. *TEN:* Toxic epidermal necrolysis.

## 6 Incidence and epidemiology

Cutaneous toxicities are among the earliest occurring adverse events, with less than a month from treatment initiation in most cases [55]. The incidence and clinical epidemiology vary widely depending on the immunotherapy modalities, the underlying malignancy, and individual patient factors [55], [56]. For ICIs, skin-related adverse events are among the most common associated toxicities, with large-scale multicenter studies reporting an incidence of around 40 % [57]. A recent meta-analysis reported a pooled estimation of ∼35 % incidence for ICI-related dermatologic irAEs, which depends on individual patient characteristics, ICI drug class, combination therapy protocol, type of treated malignancy, and duration of treatment [58]. Higher frequencies are observed in CTLA-4-containing protocols [58]. Other immunotherapy modalities have reported similar rates [59]. Additionally, combination therapies involving multiple ICIs or chemotherapy may also increase the risk of developing a rash [60], [61], [62]. Maculopapular rash and pruritus are the most prevalent manifestations, while less common presentations include vitiligo-like depigmentation, lichenoid eruptions, and psoriasiform dermatitis (Table 2). Certain toxicities, such as vitiligo-like depigmentation and hypopigmentation, are more frequent in melanoma patients, reflecting the heightened immune response against melanocytic antigens, as around 3.5 % of patients undergoing immunotherapy for melanoma have developed vitiligo in pooled analyses [63], [64]. Rare but severe toxicities like bullous pemphigoid or Stevens-Johnson Syndrome occur in very few cases but carry significant morbidity.

**Fig. 1:**
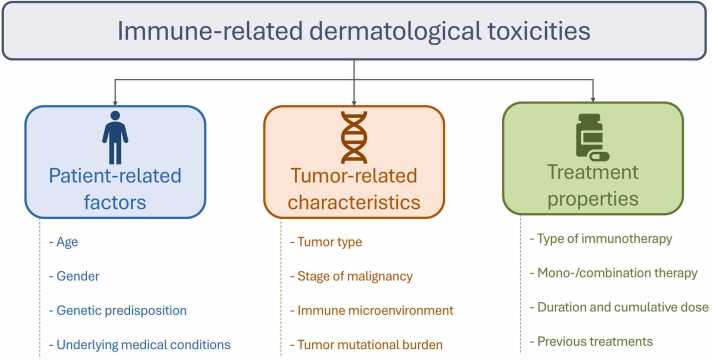
Factors affecting the occurrence of immune-related dermatological toxicities.

Epidemiological patterns suggest an earlier onset for mild to moderate toxicities, typically within weeks of treatment initiation, whereas severe manifestations may have a delayed presentation [65]. Factors such as patient age, sex, genetic predisposition, and prior history of autoimmune diseases may influence susceptibility and presentation, although the current evidence is inconclusive [62], [66]. Female gender, younger age, and certain malignancies, such as melanoma, have been associated with a higher risk of developing a rash. Additionally, pre-existing skin conditions, such as atopic dermatitis, psoriasis, or lupus erythematosus, may predispose patients to more severe dermatologic toxicities [67], [68]. Fig. 1 illustrates the factors involved in the immune-related dermatological toxicities’ risk of occurrence.

## 7 Management strategies

Management of dermatological immune-related toxicities often requires a multimodal approach, guided by the severity of symptoms per CTCAE grading [69]. Mild toxicities, such as localized rash or pruritus, are generally managed with symptomatic treatments, including topical corticosteroids, emollients, and antihistamines, without the need for interrupting immunotherapy [70]. Topical corticosteroids show anti-inflammatory and immunosuppressive effects by inhibiting cytokine production, reducing immune cell infiltration, and suppressing keratinocyte activation [71], [72]. High-potency corticosteroids may be indicated for severe or widespread symptoms, while lower-potency formulations are suitable for milder presentations or sensitive areas of the skin [73]. Antihistamines, particularly H1 receptor antagonists, are effective in relieving pruritus and urticaria by blocking the action of histamine, a key mediator of allergic and inflammatory responses. Due to their non-sedating properties, second-generation antihistamines may be a preferred choice for long-term management [74]. The choice of topical corticosteroid and antihistamine formulation depends on the severity, location, and type of dermatologic adverse event, as well as patient preferences and comorbidities. Gabapentin or aprepitant are second-line choices for refractory pruritus [75], [76]. Close monitoring for adverse effects, such as skin atrophy or sedation, is necessary with prolonged or high-dose corticosteroid and antihistamine use. Additionally, patient education regarding proper application techniques, potential side effects, and adherence to treatment regimens is essential for optimizing therapeutic outcomes.

Oral corticosteroids, such as prednisone or methylprednisolone, are commonly used as first-line therapy for more significant cutaneous manifestations [73]. However, long-term use of corticosteroids is associated with significant adverse effects, including metabolic disturbances and osteoporosis. Moreover, recent studies have shown the inverse impact of peak dose corticosteroid administration and second-line immunosuppressants on the survival of cancer patients with dermatological irAEs [77]. Considering the chief role of immunosuppression in managing these toxicities, alternative administration protocols, such as dose-splitting, should be considered, especially since the cumulative corticosteroid dose does not affect the patients’ survival.

For persistent non-severe cases, temporary discontinuation of the immunotherapy might be considered. Severe toxicity (CTCAE grade 3) requires a same-day dermatology consult, along with more intensive measures, including systemic corticosteroids at immunosuppressive doses, often combined with adjunctive therapies, including immunomodulators such as intravenous immunoglobulin (IVIG) and disease-modifying anti-rheumatic drugs (DMARDs) such as calcineurin inhibitors, for refractory cases [78], [79]. Conventional synthetic DMARDs, such as azathioprine, methotrexate, and mycophenolate mofetil, offer alternatives to corticosteroids for long-term maintenance therapy or steroid-sparing effects. These immunomodulators suppress immune cell proliferation and cytokine production, thereby reducing inflammation and preventing disease flares. Biologic DMARDs, including TNF-α inhibitors (such as infliximab and etanercept), interleukin-12/23 inhibitors (such as ustekinumab), and interleukin-17 inhibitors (such as secukinumab and brodalumab), target specific pathways involved in the pathogenesis of dermatologic adverse events, offering targeted and effective therapy with favorable safety profiles [78], [80]. In life-threatening conditions (CTCAE grade 4), such as SJS/TEN, immediate discontinuation of immunotherapy, hospitalization, and multidisciplinary management in an intensive care setting are essential [81]. Early intervention in a specialized burn or intensive care unit is critical to improve outcomes. In such cases, systemic immunosuppressive therapies, including intravenous corticosteroids or immunoglobulins, may be employed. Long-term management focuses on addressing chronic sequelae, such as scarring, persistent pruritus, or pigmentation changes, often requiring dermatological expertise. Proactive strategies, including early diagnosis, patient education on skin care, and close monitoring during therapy, are essential to minimize complications and optimize outcomes without compromising cancer treatment efficacy [82].

## 8 Prognostic implications

Dermatologic toxicities may serve as potential prognostic indicators of treatment outcomes. Several studies have suggested that the occurrence and severity of dermatologic toxicities during immunotherapy correlate with treatment efficacy and survival outcomes. A recent meta-analysis by Han et al. found that patients who developed immune-related skin toxicities, such as rash or pruritus, had significantly improved overall survival [83]. A recent multicenter retrospective cohort showed that ICI-treated cutaneous malignancies have consequent survival advantages [84]. Similarly, in patients with melanoma treated with ICIs, the development of cutaneous adverse events, including vitiligo and rash, was associated with better response rates and prolonged survival [85]. Latest studies have reported a lower mortality risk and around 50 % higher response rate in immunotherapy-treated lung cancer patients with dermatologic toxicities [86]. Vitiligo-like depigmentation is significantly related to improved survival outcomes, with almost four times improved overall survival in melanoma patients developing vitiligo post-immunotherapy [64]. Meanwhile, limited studies have reported controversial findings, with non-vitiligo dermatological toxicities being associated with shorter overall survival in melanoma patients undergoing immunotherapy [87].

The association of melanoma vitiligo-like depigmentation is considered a consequence of an immune response to matching antigens among melanocytes and melanoma cells [88]. Studies have suggested the role of melanocyte-specific T-cell responses in the pathogenesis of immunotherapy-induced vitiligo, as, for instance, T-cells that recognize common melanocyte antigens, such as Melan-A and gp100, have been detected in the peripheral blood and skin lesions of patients with melanoma-associated vitiligo [89]. However, genetic predisposition, tumor-specific factors, and host immune profiles may affect the susceptibility and severity of depigmentation reactions following immunotherapy [88], [90].

Conversely, severe or persistent dermatologic toxicities, such as bullous disorders or SJS/TEN, may indicate immune hyperactivation and systemic inflammation, potentially leading to treatment interruptions or discontinuations and adverse prognostic implications [91]. Moreover, the timing and onset of dermatologic toxicities may provide insights into treatment response and disease progression. Early-onset cutaneous adverse events, occurring within the first few weeks to months of initiating immunotherapy, have been associated with favorable treatment outcomes and durable responses, suggesting robust antitumor immunity and effective drug activity, while late-onset or delayed toxicities may indicate immune escape mechanisms or acquired resistance, warranting close monitoring and reassessment of treatment strategies [92].

## 9 Predictive biomarkers

Autoantibodies, blood cell counts and ratios, cytokine profiles, and human leukocyte antigen (HLA) alleles and genetic polymorphisms have been proposed as potential predictive biomarkers for the development of dermatologic toxicities associated with various therapeutic agents, including cancer immunotherapy [93]. Inherited genetic variations can influence both the immune system’s response to treatment and the severity of adverse events. Mutations in key genes, such as TP53 in Li-Fraumeni syndrome or HLA alleles associated with immune regulation, may predispose patients to more severe or atypical toxicities, potentially necessitating treatment adjustment or discontinuation. For instance, patients with Li-Fraumeni syndrome are typically considered to be more resistant to immunotherapy, since the mechanisms of tumor immunogenicity are compromised, which affects both the efficacy of treatment and the occurrence of irAEs [94], [95].

Cytokines and acute-phase proteins are key markers of systemic inflammation and immune dysregulation, contributing to the development of dermatologic toxicities. Elevated levels of pro-inflammatory cytokines, such as TNF-α, IL-6, and IL-1β, have been implicated in the pathogenesis of rash, pruritus, and other cutaneous adverse events associated with immunotherapy and targeted therapies [96], [97]. Genetic variants in genes encoding inflammatory mediators and immune regulators also influence susceptibility to dermatologic toxicities by modulating immune responses and inflammatory pathways. Single nucleotide polymorphisms in genes involved in cytokine signaling, such as TNF-α and IL-6, have been implicated in the development of rash, pruritus, and other cutaneous adverse events in patients treated with immunotherapy and targeted agents [98]. Moreover, changes in systemic inflammatory markers during treatment may serve as dynamic biomarkers for monitoring disease activity, treatment response, and disease progression. For instance, changes in serum levels of C-reactive protein and IL-6 have been correlated with the onset and resolution of dermatologic toxicities in cancer patients receiving immunotherapy [99].

HLA alleles play a central role in immune recognition and response by presenting antigens to T-cells and regulating immune cell activation. Certain HLA alleles have been implicated in the pathogenesis of immune-related adverse events, including dermatologic toxicities, by influencing immune tolerance, antigen presentation, and T-cell activation [100]. For instance, specific HLA class I alleles, such as HLA‐B*57:01, have been associated with an increased risk of SJS/TEN in patients treated with certain medications, such as abacavir and carbamazepine [101], [102]. However, more studies are required to determine the prognostic role of these factors as potential biomarkers.

## 10 Gut microbiome and gut-skin axis

The gut microbiome plays a significant role in immune regulation and systemic inflammation, thereby influencing the development and severity of dermatologic toxicities associated with therapeutic interventions [103], [104]. Recent studies have accordingly suggested a strong role of gut microbiome modulation in immune regulation and immunotherapy response [105]. Evidence from animal models and human studies suggests a significant interplay between gut microbiota composition, host immune responses, and dermatologic adverse events, highlighting the potential for microbiome modulation as a therapeutic strategy [106]. Gut microbiota predicts the clinical outcome and objective response to immunotherapy, along with the potential to predict the irAEs [107]. For instance, studies have suggested that the pathobionts are more abundant in patients with severe irAEs [108]. Specifically, a higher prevalence of some groups, such as *Streptococcus* and *Stenotrophomonas,* is observed in patients with severe irAEs—which varies among studies [109]. Restoration of gut microbiota diversity through fecal microbiota transplantation or probiotic supplementation also affects irAEs and has shown effectiveness in improving treatment outcomes in preclinical models [110]. Additionally, there have been efforts to introduce predicting models for irAEs based on the intestinal microbiome composition [111].

Apart from the intestinal microbiome, the skin microbiome is also directly associated with cutaneous toxicities in cancer patients [25], [112]. Moreover, preclinical studies have also highlighted the impact of skin damage on deranging intestinal homeostasis and modifying gut microbiome [113]. Mechanistic insights into the gut-skin axis have revealed that gut microbiota-derived metabolites and microbial antigens can modulate immune responses and inflammation within the skin as a key modifier of antitumor immune response [114], [115]. Short-chain fatty acids, such as butyrate and propionate, produced by gut bacteria have anti-inflammatory effects and may affect cutaneous inflammation and toxicity [116]. Conversely, dysbiosis-associated microbial antigens and pro-inflammatory mediators may exacerbate dermatologic adverse events by promoting immune dysregulation and tissue damage [117].

## 11 Limitations and future directions

Despite the latest advancements in understanding immune-related dermatological toxicities, several limitations remain in our current knowledge, which are reflected in this review. First, the pathophysiology of these toxicities remains incompletely understood, the most important being the precise molecular and cellular mechanisms underlying individual variability in immune responses. While ICIs have been broadly studied, the lack of predictive biomarkers on the onset, severity, and duration of dermatological toxicities with desirable clinical applicability is still a major gap to address. Additionally, most clinical data are derived from clinical trials, which may not fully represent real-world treated populations containing diverse demographics, comorbidities, and concurrent therapies. This patient selection bias, along with reporting/observer bias would ultimately result in underreporting or misclassification of toxicities. Moreover, the variability observed in grading dermatological toxicities in some former case reports and small-scale studies adds to the limitations in the applicability of findings. The role of genetic predisposition also requires further studies to establish solid associations. Furthermore, the impact of environmental factors, such as microbiome composition, is not yet well-defined, and the long-term follow-up data on the chronic effects of these toxicities are limited.

Future directions in understanding and managing immunotherapy-related dermatological toxicities should focus on advancing early detection, improving therapeutic interventions, and enhancing patient quality of life. Biomarker discovery is promising for identifying patients at higher risk of developing dermatological toxicities. Innovations in therapeutic approaches, such as the use of targeted immunomodulators, could prevent further toxicities, while preserving the antitumor efficacy of immunotherapy interventions. Developing algorithms to predict the onset and severity of toxicities through artificial intelligence and machine learning could facilitate clinical decision-making. Longitudinal studies on the chronic and long-term sequelae of dermatological toxicities are essential for understanding their impact on survivorship and designing comprehensive care plans. More preclinical and clinical studies are required to determine the role of gut microbiome in the pathogenesis and treatment of dermatologic toxicities. Finally, interdisciplinary collaboration between oncologists, dermatologists, and palliative care specialists, is critical in addressing the complexities of these toxicities, ultimately ensuring that the benefits of immunotherapy are maximized with minimal compromise to patient safety and quality of life.

## 12 Conclusion

Dermatologic toxicities are among the most common irAEs and represent significant clinical challenges, requiring close monitoring and patient-centered management approaches. Further preclinical and clinical studies are required to better understand the underlying mechanisms, introduce potential predictive biomarkers, and optimize treatment strategies to minimize the impact of these cutaneous adverse events on patients’ quality of life and clinical outcomes.

## Ethics approval and consent to participate

Not applicable.

## Funding

None.

## Consent for publication

Not applicable.

## CRediT authorship contribution statement

**Akbarzadeh Mohammad Amin:** Writing – original draft, Investigation, Data curation, Conceptualization. **Vaez-Gharamaleki Yosra:** Writing – original draft, Methodology, Investigation, Data curation. **Hosseini Mohammad-Salar:** Writing – original draft, Visualization, Methodology, Investigation, Data curation, Conceptualization. **Sanaie Sarvin:** Writing – review & editing, Visualization, Supervision, Conceptualization. **Mahmoodpoor Ata:** Writing – review & editing, Methodology. **Jadidi-Niaragh Farhad:** Writing – review & editing, Investigation, Data curation.

## Declaration of Competing Interest

The authors declare that they have no known competing financial interests or personal relationships that could have appeared to influence the work reported in this paper.

## Data Availability

No data was used for the research described in the article.
